# Effects of Preparation Methods on the Microstructure and Mechanical Properties of Graphene-Reinforced Alumina Matrix Composites

**DOI:** 10.3390/ma15155445

**Published:** 2022-08-08

**Authors:** Danxia Zhang, Xiaoqian Wu, Bi Jia, Hanmei Jiang, Yin Liu, Rong Wang, Qian Yang, Huiming Wu, Chunyan Wu

**Affiliations:** Chongqing Key Laboratory of Nano-Micro Composite Materials and Devices, School of Metallurgy and Materials Engineering, Chongqing University of Science and Technology, Chongqing 401331, China

**Keywords:** alumina-graphene composites, preparation methods, microstructure, mechanical properties

## Abstract

Recent years have witnessed a growing research interest in graphene-reinforced alumina matrix composites (Al_2_O_3_-G). In this paper, to better achieve the dispersion of graphene in composites, a ball milling method for adding raw materials step by step, called stepwise feeding ball milling, was proposed. The Al_2_O_3_-1.0 wt % graphene composites were prepared by this stepwise feeding ball milling and hot pressing. Then, the effects of sintering temperature and sintering pressure on the microstructure and mechanical properties of composites were studied. Results showed that the bending strength, fracture toughness and Vickers hardness of composites increased firstly and then decreased with increasing sintering temperature. The mechanical properties of composites were all at their maximum with the sintering temperature of 1550 °C. For example, the bending strength of composites reached 754.20 MPa, which was much bigger than 478.03 MPa at 1500 °C and 364.01 MPa at 1600 °C. Analysis suggested that the strength of composites was mainly related to the grain size, microflaw size and porosity.

## 1. Introduction

Al_2_O_3_ ceramic is one of the most promising and progressive structural materials, which has high wear resistance, corrosion resistance, high-temperature resistance and oxidation resistance [1,2,3,4,5]. Al_2_O_3_ ceramic has a wide range of applications in advanced manufacturing, aerospace, defense industry, integrated circuit manufacturing, deep-sea exploration and other fields. However, the inherent brittleness of Al_2_O_3_ seriously affects their application in the field of engineering structures. In recent years, graphene as a two-dimensional material has been widely used as a reinforcing material in the toughening of ceramics [6,7,8,9]. For example, Cygan et al. prepared Al_2_O_3_-G by spark plasma sintering (SPS), and studied the mechanical properties of composites [10]. They reported that the fracture toughness of composites with series of contents of multilayered graphene and graphene oxide was in a range of 4.56 MPa·m^1/2^ and 4.33 MPa·m^1/2^ [10]. Celik et al. prepared Al_2_O_3_-G by SPS, and studied the effects of microstructure on the mechanical properties of materials [11]. They reported that the 3 vol. % graphene platelets addition into monolithic Al_2_O_3_ caused fracture toughness to increase by 26.7% (reaching 3.8 MPa·m^1/2^) [11]. Porwal et al. prepared Al_2_O_3_-G with different graphene contents by SPS [12]. Yazdaniet al. prepared Al_2_O_3_-G by using SPS and hot-press sintering (HP), and further studied the effects of two different sintering methods on the grain sizes and mechanical behavior of composites [13]. They found that both types of composites obtained a high bending strength and fracture toughness of >400 MPa and 5.5 MPa·m^1/2^ [13].

Recent years witnessed a growing research interest in preparation and mechanical properties of Al_2_O_3_-G. However, the graphene is quite difficult to be uniformly dispersed in the Al_2_O_3_ matrix, which has a significant effect on the microstructure and mechanical properties of materials. The dispersion of graphene in the Al_2_O_3_ matrix is still a challenging work. Some work has been focused on the further optimization of the synthesis method of Al_2_O_3_-based composites [14]. In this work, to better achieve the dispersion of graphene in composites, we proposed a ball milling method for adding raw materials step by step, called stepwise feeding ball milling. Through this novel method, we achieved uniform dispersion of graphene in an alumina matrix, and prepared Al_2_O_3_-G having good performances by HP. The fracture strength and fracture toughness of composites reached a great high value of 754.20 MPa and 7.50 MPa·m^1/2^, respectively. Then, the effects of the ball milling method, sintering temperature and sintering pressure on the microstructure and mechanical properties of composites were studied. The main influencing mechanisms of material properties were discussed.

## 2. Materials and Methods

### 2.1. Materials

The Al_2_O_3_ powder (Hangzhou Wanjing New Materials Co., Ltd., Hangzhou, China, 0.5 μm, ≥99.0%) and graphene powder (Wuxi Nadun Technology Co., Ltd., Wuxi, China, 1 μm, ≥95.0%) were used as raw materials. Zirconia balls (≥99.0%) with a diameter of 50 mm were purchase from Zhengzhou SKY Universe Trade Co., Ltd., Zhengzhou, China, Absolute alcohol (Wuxi Nadun Technology Co., Ltd., Wuxi, China, ≥99.7%) was used as a dispersing agent in ball milling. All chemicals were used as received without further purification.

### 2.2. Fabrication of Al_2_O_3_-G by One-Step Feeding Method

Firstly, graphene material was mixed with the Al_2_O_3_ powder in a 1:99 weight ratio. The mixture powders and zirconium balls (1:2 weight ratio) were ball-milled in absolute ethanol at 90 rpm min^−1^ for 72 h to get a slurry. Then, the slurry was dried at a temperature of 50 °C for 12 h and sieved through an 80-mesh sieve. Finally, the prepared powders were loaded into a graphite grinding tool, and then were hot-pressed at 1550 °C, 40 MPa for 60 min to gain the composites, named as Al_2_O_3_-G-O.

### 2.3. Fabrication of Al_2_O_3_-G by Stepwise Feeding Method

To enhance the desperation of graphene, we proposed a novel multistep feeding method to gain the composites, called the stepwise feeding method. In details, the 1.0 wt % graphene powder was firstly added into the ball mill, followed by Al_2_O_3_ powder and zirconia balls in a 1:2 weight ratio. The mixed powders were milled in 200 g absolute ethanol at 90 rpm min^−1^, and then 200 g of ethanol were continued to be added after ball milling for 5 h; then another 400 g ethanol were added after ball milling for 4 h. Until the mixed powders presented a paste state, another 400 g ethanol were added, and then the mixture was continued to be ground for 40 h to obtain the slurry. After grinding, the slurry was dried at a temperature of 50 °C for 12 h, and then was sieved through a 80-mesh sieve. Finally, the prepared powders were hot-pressed at 1550 °C, 40 MPa for 60 min to gain the composites, named as Al_2_O_3_-G-M.

### 2.4. Material Characterization

The relative densities of Al_2_O_3_-G composites were measured by the Archimedes method with deionized water as the immersing medium. In order to determine their relative density, the theoretical density of the nanocomposites was calculated by the volume-based rule of mixtures, assuming densities of 3.96 g/cm^3^ and 2.1 g/cm^3^ for Al_2_O_3_ and graphene, respectively [6].

The microstructure, fracture morphology, and interface bonding of etch composites were observed by scanning electron microscopy (SEM; JSM-7800F, JEOL Co., Ltd., Tokyo, Japan; at 10 kV). The compositions and elemental distributions of the Al_2_O_3_-G composites were analyzed using an energy dispersive X-ray spectrometry (EDS; JEOL Co., Ltd., Tokyo, Japan). All samples were prepared for microscopy by cutting cross sections parallel to the hot-pressing direction and then polishing to a 0.30 μm finish using diamond abrasives. The phase composition of the Al_2_O_3_-G composites was analyzed using X-ray diffraction (XRD; XRD-7000S/L, Shimadzu, Tokyo, Japan). The average grain size of composite was measured by Image J (Image J; US National Institutes of Health, Bethesda, MD, USA) by counting a minimum of 100 grains. The longest diameter of the grain was reported as the average grain size.

### 2.5. Mechanical Testing

Hardness of the samples was determined by Vickers indentation (452SVD, Shanghai Baihe Instrument Technology Co., Ltd., Shanghai, China) using the load of 10 kg and the dwell time was 15 s. Reported values were obtained from an average of 5 indentations on a single specimen.

Universal testing machine (UH6104A, Jinan Yongke Test Instrument Co., Ltd., Jinan, China) was used to check the bending strength and fracture toughness of the samples. The bending strength was characterized by the three-point bending test with 0.5 mm/min loading rate. The size of bending specimen is 3 × 4 × 35 mm, and the span is 30 mm. The fracture toughness of the samples (specimen size was 3 × 4 × 35 mm, notched size was 2 mm) was measured by the single-edge-v-notched beam (SEVNB) method.

## 3. Results and Discussion

### 3.1. Effects of Graphene Dispersion on the Microstructure of Composites

**Figure 1:**
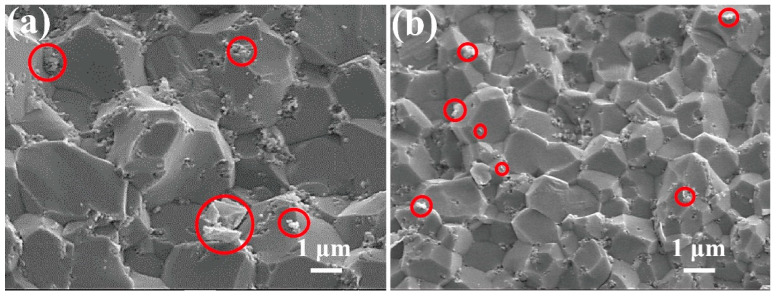
SEM images of (**a**) Al_2_O_3_-G-O, and (**b**) Al_2_O_3_-G-M (The red circle represents graphene).

Figure 1 shows micrographs of the fracture surfaces of Al_2_O_3_-G-O and Al_2_O_3_-G-M. The average grain size of Al_2_O_3_-G-O by the one-step feeding method is close to 3.29 μm (Figure 1a), and the graphene is seriously agglomerate in Al_2_O_3_-G-O. As a contrast, there is almost no agglomeration of graphene in Al_2_O_3_-G-M by stepwise feeding ball milling, and the average grain size is reduced to 2.39 μm. In addition, this grain size is also much smaller than 4.2 μm of the Al_2_O_3_-G obtained in the literature [13]. This indicates that the graphene disperses more uniformly in ceramic via stepwise feeding ball milling, which could inhibit grain growth of Al_2_O_3_ and hinder movement of grain boundaries resulting in a finer microstructure [7]. The mixing movement of powders of different compositions and properties is a very complex chaotic process. It is worth noting that the effect of the absolute ethanol on the dispersion of graphene will affect the graphene-reinforced alumina matrix composites. Obviously, the average size of graphene in Al_2_O_3_-G-M by stepwise feeding ball milling is much smaller than that in Al_2_O_3_-G-O by the one-step feeding method, indicating that stepwise feeding ball milling is more beneficial to improve the dispersion of graphene in absolute ethanol. This is probably because stepwise feeding ball milling could achieve the higher concentration of graphene in absolute ethanol, which could improve the affinity of the solvent molecules for graphene and enhance the dispersion of graphene in alumina matrix composites [15,16].

**Figure 2:**
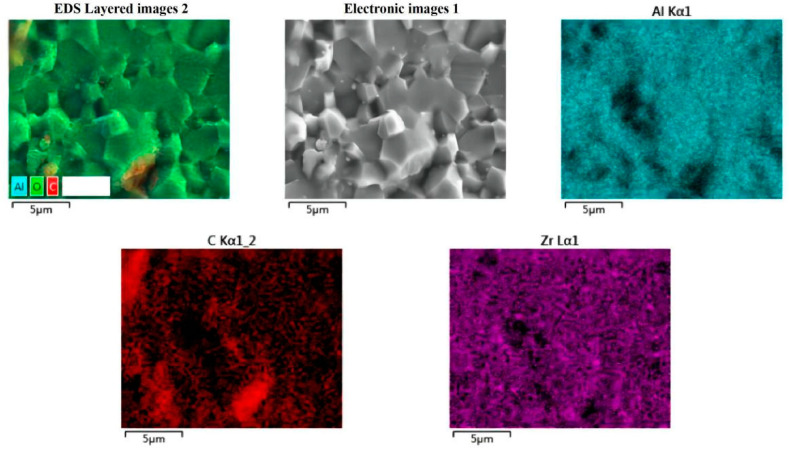
EDS images of Al_2_O_3_-G-M.

Moreover, the element distribution of Al_2_O_3_-G-M is also measurement. As shown in Figure 2, the C element disperses evenly in Al_2_O_3_-G-M, demonstrating that the stepwise feeding ball milling could result in the good dispersion of graphene. In addition, a small amount of Zr element is also found in the ceramic, which may be an impurity introduced by the zirconium grinding ball.

### 3.2. Effects of Sintering Temperatures on the Microstructure of Composites

**Figure 3:**
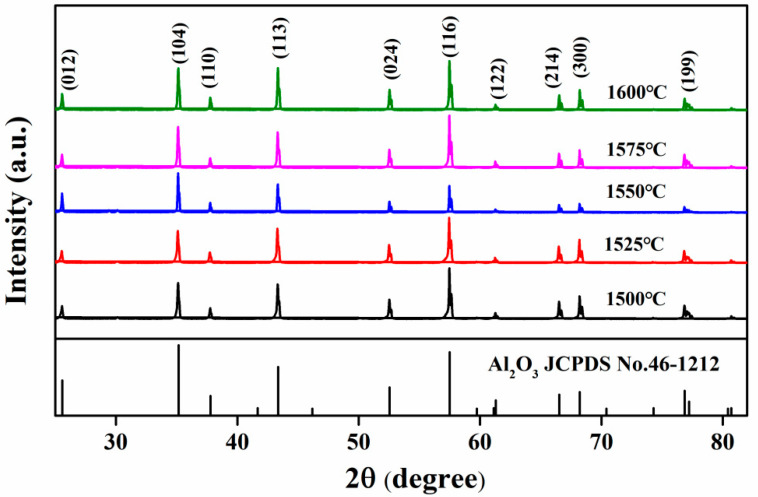
XRD patterns of Al_2_O_3_-G-M hot-pressed at different sintering temperature.

The XRD diffraction patterns of Al_2_O_3_-G-M composites hot-pressed at different sintering temperatures are shown in Figure 3. The diffraction peaks of all samples at 2θ are 25.8°(012), 35.2°(104), 37.8°(110), 43.4°(113), 52.5°(024), 61.1°(116), 66.5°(122), 61.1°(214), 66.5°(300), 77.2°(199), consistent with α-Al_2_O_3_ (corundum) (JCPDS No. 46-1212) [17]. As the HP sintering temperature increases from 1500 °C to 1600 °C, the crystal phase of the composite material is still α-Al_2_O_3_ (corundum), indicating that the HP sintering temperature has no significant effect on the crystal phase of the ceramic composite and does not change the crystal phase of the alumina matrix. In addition, because the content of graphene is too small (about 1.0 wt %) and is evenly dispersed, there is no graphene diffraction peak in Al_2_O_3_-G-M. 

### 3.3. Effect of Feeding Methods on the Mechanical Properties of Composites

The relative density, bending strength, fracture toughness and Vickers hardness of Al_2_O_3_-G-M graphene composites prepared by different feeding methods during ball milling are shown in Table 1. It is very obvious that the fracture strength and fracture toughness of composites corresponding to the stepwise feeding ball milling are much higher, and their values reach 754.20 MPa and 7.50 MPa·m^1/2^. This value is much bigger than 4.56 MPa·m^1/2^ [10], 3.8 MPa·m^1/2^ [11] and 5.5 MPa·m^1/2^ [13] of the composites obtained in the literature. This shows that the quality of graphene dispersion has great influence on the mechanical properties of the composites.

### 3.4. Effects of Sintering Temperatures on Mechanical Properties of Composites

**Figure 4:**
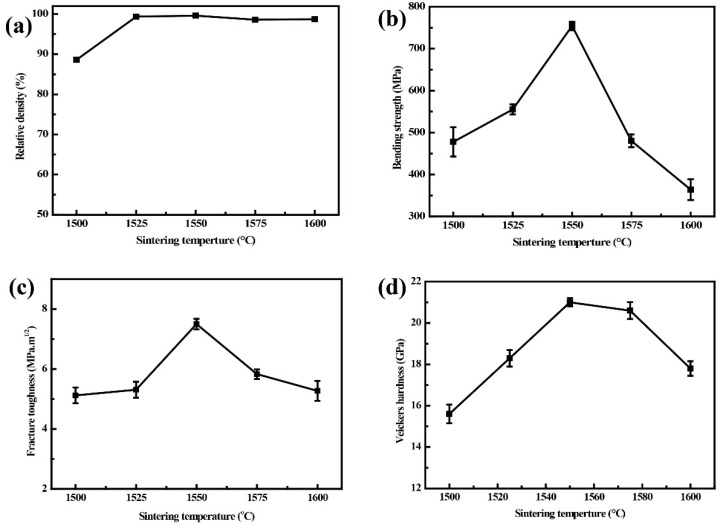
Changing trends of (**a**) relative density, (**b**) bending strength, (**c**) fracture toughness, and (**d**) Vickers hardness of Al_2_O_3_-G-M hot-pressed at different sintering temperatures.

Figure 4 shows the effects of sintering temperature on the relative density, bending strength, fracture toughness, and Vickers hardness of Al_2_O_3_-G-M. As we know, adding graphene, known as a 2D material, can well improve the densification of the materials. As can be seen from Figure 4a, the relative density of composites prepared at 1500 °C is low, which is only 88.59%. When the sintering temperature increases to 1550 °C, the relative density of composites reaches 99.60%. With the further increase of the sintering temperature, the relative density of composites tends to be stable and remains above 98%. Figure 4b–d shows that the bending strength, fracture toughness and Vickers hardness of Al_2_O_3_-G-M all increase firstly and then decrease with increasing sintering temperature. The changing trends of the mechanical properties of composites with the sintering temperature are consistent with those reported in the literature, and there is a sintering temperature corresponding to the best properties of composites [10,11,12,13]. In this work, when the sintering temperature is 1550 °C, the mechanical properties of composites are all at their maximum. For example, the bending strength of composites reaches 754.20 MPa, which is much bigger than 478.03 MPa at 1500 °C and 364.01 MPa at 1600 °C. The fracture toughness of material is also effectively improved, reaching 7.50 MPa·m^1/2^. In the sintering temperature range of 1500 °C to 1550 °C, the Vickers hardness of composites increased from 15.6 GPa to 21.00 GPa. While the Vickers hardness of composites decreased to 17.8 GPa at 1600 °C.

**Figure 5:**
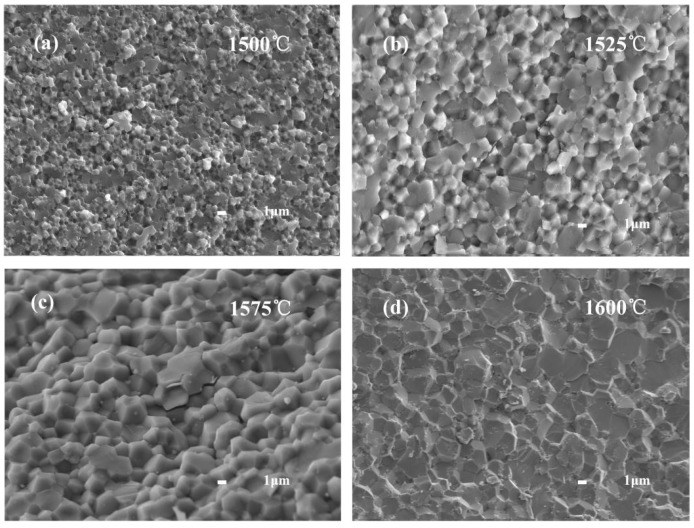
SEM images of the microstructure of the fracture surfaces of Al_2_O_3_-G-M hot-pressed at different sintering temperatures: (**a**) 1500 °C; (**b**) 1525 °C; (**c**) 1575 °C; (**d**) 1600 °C.

Figure 5 shows the SEM images of the microstructure of the fracture surfaces of Al_2_O_3_-G-M hot-pressed at different sintering temperature. As can be seen from Figure 5a,d, the significant micropores appear in the microstructure of composites corresponding to the sintering temperatures of 1500 °C and 1600 °C. Figure 5b,c shows the obvious microflaws in the microstructure of composites corresponding to the sintering temperatures of 1525 °C and 1575 °C, while in the microstructure of composites with sintering temperature of 1550 °C (Figure 1b), we do not observe the obvious microflaws and micropores. As we know that when the sintering temperature is low, the densification is not enough, and there are obvious microflaws/micropores with a size larger than the grain size, while if the sintering temperature is too high, it is easy to cause abnormal growth of the grains and produce the obvious microflaws. The average grain sizes and maximum grain sizes of composites with different sintering temperatures are shown in Table 2.

According to the classical Griffith fracture theory, the fracture strength of brittle materials can be expressed as [18]

(1) $$\sigma_{f}=\sqrt{\frac{2E\gamma }{\pi a}}$$

where *E* is the Young’s modulus of materials; *γ* is the fracture surface energy of materials; *a* is the critical flaw size of materials. The studies showed that the critical flaw size of ceramics is related to the grain size and the size of microflaw around the grain [19,20]. Then, Equation (1) can be modified as

(2) $$\sigma_{f}=\sqrt{\frac{2E\gamma }{\pi \left(R+s\right)}}$$

In Equation (2), *R* is the grain size, and *s* is the size of flaw around the grain. Meanwhile, the preexisting microflaws and micropores could reduce the densification of materials, and then reduce the Young’s modulus of materials, followed by the decreasing of the fracture strength of materials [21]. It can be concluded that the fracture strength of materials would decrease with increasing grain size, microflaw size and porosity. Based on the above analysis of microstructures of composites with different sintering temperatures and Equation (2), it can be concluded that the strength of composites with a sintering temperature of 1550 °C should be highest. This is completely consistent with the experimental phenomenon. The fracture toughness and hardness of materials are also related to the sizes of big grains, preexisting microflaws and micropores [22,23,24]. Figure 5 also indicates that there is no obvious difference in the toughening mechanism of Al_2_O_3_-1.0 wt % graphene composites prepared at different sintering temperatures. The increase of interfacial bonding strength caused by the addition of graphene, the pull-out of graphene and the deflection during crack propagation will all improve the fracture toughness of the composites [10,11,12,13].

### 3.5. Effects of Sintering Pressure on Mechanical Properties of Composites

**Figure 6:**
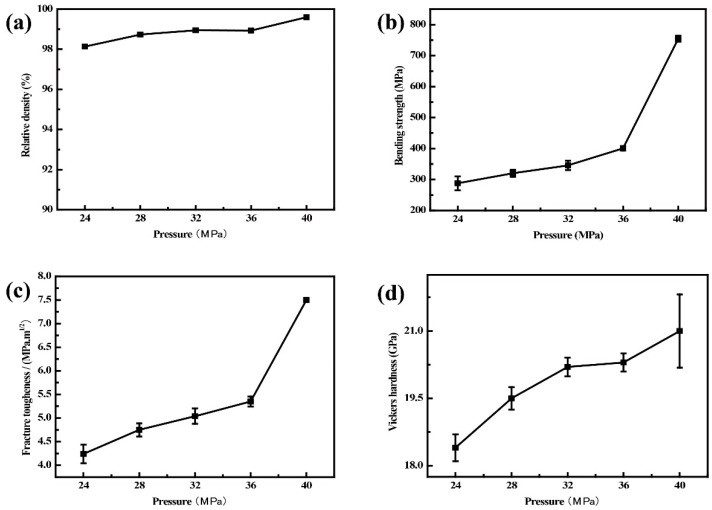
Changing trends of (**a**) relative density, (**b**) bending strength, (**c**) fracture toughness, and (**d**) Vickers hardness of Al_2_O_3_-G-M hot-pressed at different sintering pressure.

Figure 6 shows the effects of sintering pressure on the relative density, bending strength, fracture toughness, and Vickers hardness of Al_2_O_3_-G-M. All values increase with increasing sintering pressure, especially for the fracture strength and toughness of composites. As the sintering pressure increases from 24 MPa to 40 MPa, the bending strength of composites increases from 287.85 MPa to 754.20 MPa, and the fracture toughness of composites increases from 4.24 MPa·m^1/2^ to 7.5 MPa·m^1/2^. This is because, under greater sintering pressure, the accumulation of particles is relatively tight, and the mutual contact points and contact area among particles are increased, which greatly promotes the mass transfer process of particle rearrangement, thereby promoting the discharge of adsorption gas, eliminating the pores in the system, healing some microflaws, and improving the relative density of the material. The grains of composites can be better refined, and the microflaws and micropores can be reduced to a greater extent. Therefore, the failure of composites requires more energy, leading to the increase of the mechanical property of composites.

## 4. Conclusions

In this work, the Al_2_O_3_-G with high fracture strength and fracture toughness of 754.20 MPa and 7.50 MPa·m^1/2^ was successfully synthesized by stepwise feeding ball milling and HP. The mechanical properties of composites increased firstly and then decreased with increasing sintering temperature. The optimum sintering temperature of 1550 °C was given. The mechanical properties of composites increased with increasing sintering pressure, especially for the fracture strength and toughness of composites. This study showed that the mechanical properties of composites are mainly controlled by the sizes of big grains, preexisting microflaws and micropores. This work would provide guidance for the preparation of alumina ceramic matrix composites with excellent performances.

## Figures and Tables

**Table 1:** The relative density and mechanical properties of Al_2_O_3_-G-O and Al_2_O_3_-G-M prepared by the two feeding methods.

Name	Feeding Method	Relative Density (%)	Bending Strength (MPa)	Fracture Toughness (MPa·m^1/2^)	Vickers Hardness (GPa)
Al_2_O_3_-G-O	one-step	98.72	434.17	5.18	18.70
Al_2_O_3_-G-M	multistep	99.60	754.20	7.50	21.0

**Table 2:** The average grain size and maximum grain size of Al_2_O_3_-G-M hot-pressed at different sintering temperatures.

Sintering Temperature/°C	Average Grain Size/ μm	Maximum Grain Size/ μm
1500	0.77	1.27
1525	1.57	3.06
1550	2.39	4.91
1575	2.50	4.97
1600	2.69	5.29
